# Assessment of soluble skin surface protein levels for monitoring *psoriasis vulgaris* in adult psoriasis patients using non-invasive transdermal analysis patch: A pilot study

**DOI:** 10.3389/fmed.2023.1072160

**Published:** 2023-03-02

**Authors:** Kadri Orro, Kristiina Salk, Kristi Abram, Jelena Arshavskaja, Anne Meikas, Maire Karelson, Toomas Neuman, Külli Kingo, Pieter Spee

**Affiliations:** ^1^Department of Chemistry and Biotechnology, Tallinn University of Technology, Tallinn, Estonia; ^2^FibroTx LLC, Tallinn, Estonia; ^3^Clinic of Dermatology, Tartu University Hospital, Tartu, Estonia; ^4^Clinic of Dermatology, Institute of Clinical Medicine, Tartu University, Tartu, Estonia; ^5^PS! Pharmaconsult, Alleroed, Denmark

**Keywords:** biomarker, transdermal analysis patch, psoriasis, inflammation, dermatology, skin care diagnostics, treatment monitoring

## Abstract

To improve the care of patients with chronic inflammatory skin conditions, such as psoriasis, there is a need for diagnostic methods that can facilitate personalized medicine. This exploratory pilot study aimed to determine whether non-invasive measurements of inflammation-related proteins from psoriatic skin can be sampled using the FibroTx Transdermal Analysis Patch (TAP) to assess disease severity and monitor pharmacodynamic changes. Ten healthy volunteers and 44 psoriasis vulgaris patients were enrolled in the exploratory pilot study. Skin surface protein measurements for healthy and lesional skin were performed using TAP. Patients’ scores of psoriasis activity and severity (PASI) were documented, and differences in the thickness of skin layers were determined using sonography. The study assessed the skin surface protein levels of psoriasis patients undergoing whole-body treatment with narrow-band UVB to evaluate whether the levels of the skin surface proteins IL-1α, IL-1RA CXCL-1/2, and hBD-1 were associated with the disease activity and severity measurements. Using TAP technology, it was observed that there were clear differences in levels of IL-1α, IL-1RA, CXCL-1/2, and hBD-1 between psoriasis lesional and non-lesional skin. In addition, a positive correlation between CXCL-1/2 and desquamation, and between CXCL-1/2 and SLEB thickness was observed. During UVB treatment, the TAP measurements revealed a clear reduction of IL-1RA, CXCL 1/2, and hBD-1 on lesional skin. Further, skin surface measurements of IL-1RA and CXCL-1/2 displayed a different profile than those achieved by visual scoring of local inflammation, thus indicating that measuring the ‘molecular root’ of inflammation appears to have value as an objective, non-invasive biomarker measurement for scoring disease severity.

## Introduction

Psoriasis is a chronic relapsing immune inflammatory dermatosis with different clinical manifestations that affects 1–3% of the world population (1, 2). Psoriasis vulgaris (PV) is the most common variant of psoriasis and is characterized by erythematous scaly plaques of the skin. It is caused by the interplay between immune cells, keratinocytes, and other skin-resident cells, mediated by adaptive and innate immune system components causing a hyperproliferation of keratinocytes and chronic inflammation in affected skin (2–6). Psoriasis patients often develop additional systemic comorbidities, such as arthritis, metabolic syndrome, diabetes, cardiovascular risk, and depression (7–9). Clinical evaluation of psoriasis is mainly performed visually using the Psoriasis Area Severity Index (PASI), a clinical score based on the assessment of the percentage of skin affected and severity of the skin erythema, induration, and desquamation (10). The PASI score allows monitoring of changes in affected skin areas over time, which may either reflect the progression, relapse, or improvement of the disease. However, several limitations of PASI have been addressed, such as not correlating the clinical extent of the disease with quality of life (11, 12) and that the PASI lacks sensitivity, as erythema, desquamation, and induration are scored with equal weight within each of the four body regions (13).

Treatment of PV depends on the severity and areas affected in patients - treatment options range from local ointments for mild psoriasis, to more harsh therapies for moderate and severe psoriasis, such as phototherapy [e.g., ultraviolet B (UVB)], photochemotherapy [e.g., psoralen ultraviolet A (PUVA)], systemic treatment with conventional agents [e.g., methotrexate, cyclosporine, and acitretin] or biological treatment [e.g., anti-tumor necrosis factor (anti–TNF-α) and interleukin inhibitors (anti-IL17, anti-IL-12/23, and anti-IL23)], where there are risks for developing side effects, such as infections (biologic therapies), or an increased risk for skin cancer (PUVA, phototherapy) (14–18). Therapeutic efficacy, defined as a diminishment in psoriasis clinical scores, does not occur instantly, and patients may not respond to therapy at all.

To improve psoriasis care, diagnostic and monitoring methods are needed that can facilitate personalized medicine. Proteins, such as interleukins, chemokines, cell surface receptors, growth factors, and anti-microbial peptides drive the biological processes underlying clinical hallmarks of psoriatic skin. We hypothesize that this psoriasis ‘molecular footprint’ may be very suitable for developing methods of detection and diagnosis of the disease, monitoring psoriasis progression, as well as measuring response to treatment.

Although skin biopsy remains one of the widely reported approaches for skin protein analyses from diseased skin (19, 20), less invasive alternatives have emerged in dermatological practice. Repetitive tape stripping has been considered a benchmark for minimally invasive methods of skin protein sampling (20–22). Further, other non-invasive methods for biomarker detection and profiling from the skin involve skin lavage (23), reverse iontophoresis (24), optical imaging (25), and a micro disk-library array patch method has also been described (26).

We have previously introduced a non-invasive method to measure soluble biomarkers directly from the skin (27). The FibroTx Transdermal Application Patch (TAP) consists of an adhesive bandage that contains a nitrocellulose insert on which specific antibodies have been printed for capturing proteins directly from the skin surface. TAP has shown the potential of detecting soluble skin surface biomarkers on healthy and irritated skin (27, 28). Further, using TAP, biomarkers on psoriatic lesions of pediatric patients receiving topical- and systemic treatment were determined without discomfort (29, 30).

The objectives of the present pilot study were to assess whether expression patterns of inflammatory proteins, IL-1α, IL-1RA, CXCL-1/2, and hBD-1, known to be involved in psoriasis, could be measured from the skin surface of adult psoriasis patients using the FibroTx TAP technology, whether the measurements correlated with the disease severity and further, to explore whether these skin surface proteins could be used to measure pharmacodynamic effects of psoriasis treatment.

## Materials and methods

### Study participants

The study was an explorative observational non-invasive study performed at the Dermatology Clinic of Tartu University Hospital in Estonia between 2015–2016, under the approval of Tallinn Medical Research Ethical (Decision No. 2551). Patients with moderate to severe PV visiting a dermatologist at Tartu University Hospital Dermatology clinic were included in the study. Patients included in the study had not received any systemic form of medical treatment and any kind of phototherapies for at least 4 weeks prior to the study and had not received any topical form of medical treatment for at least 2 weeks prior to the study. Pregnant or breastfeeding women and volunteers with a history of other skin diseases were excluded from participation. Healthy volunteers with no positive family history of psoriasis and other chronic dermatoses were serving as a control group. All participants signed informed consent prior to the study. Detailed information regarding participants is shown in Supplementary Tables S1–S3.

### Measurements of transdermal analysis patch, skin disease severity scores, and sonography

At first, 10 healthy adult volunteers and 30 adult patients with PV were enrolled in the study to determine the differences in skin surface biomarkers. TAP-captured antibody micro-arrays coated with anti-interleukin one alpha (IL-1α), anti-interleukin one receptor antagonist (IL-1RA), −anti-chemokine (C-X-C motif) ligand 1 and 2 (CXCL-1/2), and anti-human beta-defensin 1 (hBD-1) were applied onto the skin of healthy volunteers and on the non-lesional and lesional skin of psoriasis patients. TAP-captured antibody micro-arrays were incubated on the skin for 20 min. Captured IL-1α and IL-1RA, CXCL-1/2, and hBD-1 were visualized using spot-enzyme linked immunosorbent assay (ELISA), as previously described (27). In addition, PASI (the range of PASI scores is 0–72) and assessment for local scores for erythema, induration, and desquamation (0–4 scale) of lesional skin were performed for patients at the exact location of the TAP measurements. Determination of differences in the thickness of skin layers [epidermis, sub-epidermal low-echogenic band (SLEB), and dermis] between non-lesional and lesional skin of patients was carried out using DermaLab Combo from Cortex Technology according to the manufacturer’s instructions. Ultrasound imaging was conducted from the same skin area as the FibroTx TAP measurements, after TAP removal from non-lesional and lesional skin.

### Narrow-band ultraviolet B treatment

Further, 14 adult PV patients were included for monitoring skin surface biomarkers and PASI index and local scores for erythema, inflammation, and scaling response to narrow-band UVB treatment in combination with calcipotriol/betamethasone dipropionate ointment (Dovobet®) daily. Narrow-band UVB treatment was performed all together during the 10 weeks, three times a week. For the current pilot study, biomarker measurements with FibroTx TAP were performed before the first treatment (served as baseline), after 2 weeks of treatment, and after 4 weeks of treatment. Measurements with FibroTx TAP were performed on the same position on the skin at each time-points. The PASI score was determined before and after 4 weeks of treatment. In parallel to TAP measurements, visual assessment for erythema, induration, and desquamation were performed at the exact location of TAP measurements.

### Statistical analyses

All statistical tests were performed using the statistics program JASP (version 0.9.2 for macOS). For statistical analysis, the normality of the data was tested with the Shapiro–Wilk test. Statistical significance for related groups analysis was determined using matched paired Wilcoxon signed-rank test, and the Mann–Whitney non-parametrical test was applied for two unrelated groups. For correlation analysis, non-parametrical Spearman’s Rank correlation analysis was performed, and statistical significances were verified with probability value (*p*-value). Correlation coefficients were interpreted based on Cohen (31) to indicate weak (0.1), moderate (0.3), and strong (0.5) correlations. The level of statistical significance was set at 5% (*p* < 0.05).

## Results

### FibroTx transdermal analysis patch measurements revealed distinctive skin surface protein patterns between healthy and diseased skin

**Figure 1:**
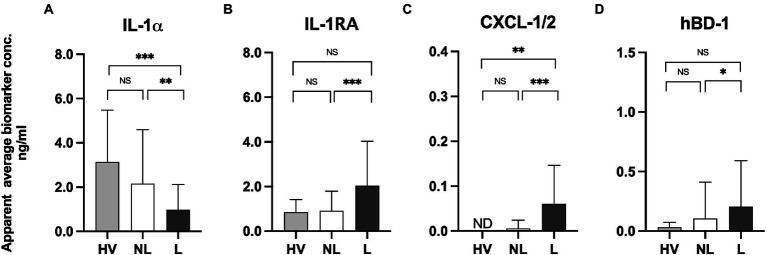
**(A)** Measurements of IL-1α, IL-1RA, CXCL-1/2, and hBD-1 on lesional and non-lesional skin of psoriasis patients and normal skin of healthy volunteers using the FibroTx TAP. The apparent average biomarker concentrations of IL-1α IL-1RA, CXCL-1/2, and hBD-1 detected from the skin surface of 10 healthy volunteers (grey bars), on non-lesional skin (NL; white bars) and lesional skin (L; black bars) of 30 psoriasis patients have been plotted. *Y*-axis: Apparent concentration of analyzed biomarker on the skin in ng/mL. *X*-axis: sampling site. Error bars on graphs present the standard deviations from the average of combined measurements of the participants. Statistical significance is indicated on panels **(A–D)**: *p < 0.05, **p < 0.01, *** *p* < 0.001; NS- not significant.

Skin surface levels of IL-1α, IL-1RA, CXCL-1/2, and hBD-1 detected on the skin of healthy volunteers appeared similar to the levels captured on the non-lesional skin of psoriasis patients (Figures 1A–D). However, notable differences were noticed in the amounts of IL-1α (3.14 vs. 0.98 ng/mL) and CXCL-1/2 (not detected vs. 0.06 ng/mL) when compared between healthy volunteers and lesional skin of psoriasis patients (*p* < 0.001 and *p* < 0.01, respectively). The trend of increase of anti-inflammatory IL-1RA and antimicrobial hBD-1 was detected on lesional skin when compared to the levels of these proteins captured on the skin surface of healthy volunteers, however, no statistical significance was observed. Comparing the levels of skin surface IL-1α, IL-1RA, CXCL-1/2, and hBD-1 captured from psoriasis patients’ lesional and non-lesional skin revealed statistically higher levels of IL-1RA (*p* < 0.001), CXCL-1/2 (*p* < 0.01), and hBD-1 (*p* < 0.05) on lesional skin (Figures 1A–D; Supplementary Figures S1A–D). However, notable lower levels of IL-1α were detected on psoriatic lesions when compared to the non-lesional skin of the same patients (*p* < 0.001).

The inverse expression patterns of IL-1α and IL-1IL-1RA on lesional and non-lesional skin of psoriasis patients, as well as the biological link between IL-1α and IL-1RA (32), prompted us to analyze the molar ratio between IL-1α and IL-1RA on lesional and non-lesional skin of psoriasis patients. IL-1α and IL-1RA bind to the same receptor, the IL-1 receptor (IL-1R), as a pro-inflammatory agonist and an anti-inflammatory antagonist, respectively. Two forms of IL-1α exist, the immature form with an MW of 31 kDa, and the mature form of 18 kDa, which are both biologically active (33). Both isoforms were recognized by antibodies used for FibroTx TAP. IL-1RA was predominantly expressed as a 17.1 kDa protein (34). The analyses revealed that there was a clear molecular excess of IL-1α over IL-1RA on non-lesional skin of psoriasis patients and the skin of healthy volunteers, regardless of whether IL-1α was present in immature or in mature form or a combination thereof. Similarly, there was a clear excess of IL-1RA over IL-1α on the lesional skin of psoriasis patients regardless of the form of IL-1α (Supplementary Table S4).

### Skin surface IL-1RA and CXCL-1/2 presented an association with edema-related sub-epidermal low-echogenic band

**Figure 2:**
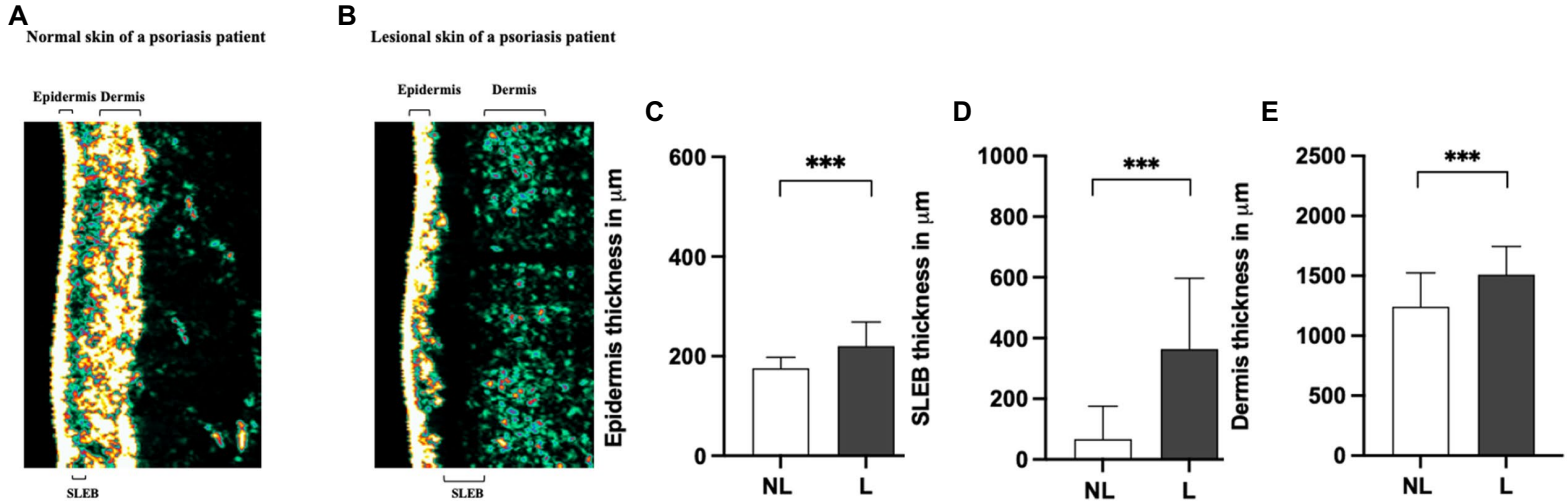
Comparison of sonography data of non-lesional and lesional skin of psoriasis patients. A representative image of the thickness of the epidermis, SLEB, and dermis in non-lesional (panel **A**) and lesional skin (panel **B**) of a psoriasis patient measured by ultrasound. 20-MHz ultrasound image of the non-lesional (panel **A**) and lesional skin area (panel **B**) of the same psoriasis patient is presented. The positions of the epidermis, SLEB, and dermis are indicated in the image. The apparent average epidermal thickness (panel **C**), SLEB thickness (panel **D**), and dermis thickness (panel **E**) analyzed from non-lesional skin (NL; white bars) and lesional skin (L; black bars) have been blotted. The ultrasound measurements are performed at the exact lesion and healthy apparent skin of the psoriasis patient where the FibroTx TAP measurements were performed and local clinical scores by the physician were stated. *Y*-axis: Average thickness of the epidermis, SLEB, and dermis, respectively, in μm. *X*-axis: sampling site. Error bars on graphs present the standard deviations from the average of combined measurements of the patients (*N* = 30). Statistical significance was determined with paired sample Wilcoxon signed-rank test (*p < 0.05, **p < 0.01, ***p < 0.001).

To determine whether differences in the molecular expression patterns of IL-1α, IL-1RA, CXCL-1/2, and hBD-1, between non-lesional and lesional skin sites of psoriasis patients associated with alterations in physical properties of skin layers, FibroTx TAP measurements of these four proteins were correlated with ultrasound measurements. First, a clear and statistically significant thickening of the epidermis (*p* < 0.001), SLEB (*p* < 0.001), and dermis (*p* < 0.001) was measured in lesional skin of psoriasis patients in comparison with non-lesional skin from the same patients (Figures 2A–D).

Correlating FibroTx TAP measurements of IL-1α, IL-1RA, CXCL-1/2, and hBD-1 and ultrasound measurements of non-lesional skin and lesional skin from the same patients revealed a positive correlation between IL-1RA and SLEB thickness (*r =* 0.45*, p =* 0.013, Supplementary Table S5A) for non-lesional skin, and strong positive correlation between CXCL-1/2 and SLEB thickness on lesional skin (*r =* 0.512*, p =* 0.004, Supplementary Table S5B).

Combining ultrasound measurements of the epidermis, dermis, or SLEB with the local scores for erythema, inflammation, and scaling of the patients did not reveal any significant correlations except for a significant positive correlation between SLEB thickness and induration (*r* = 0.402, *p =* 0.028, Supplementary Table S5C).

### Skin surface proteins were normalizing over narrow-band ultraviolet B therapy

**Figure 3:**
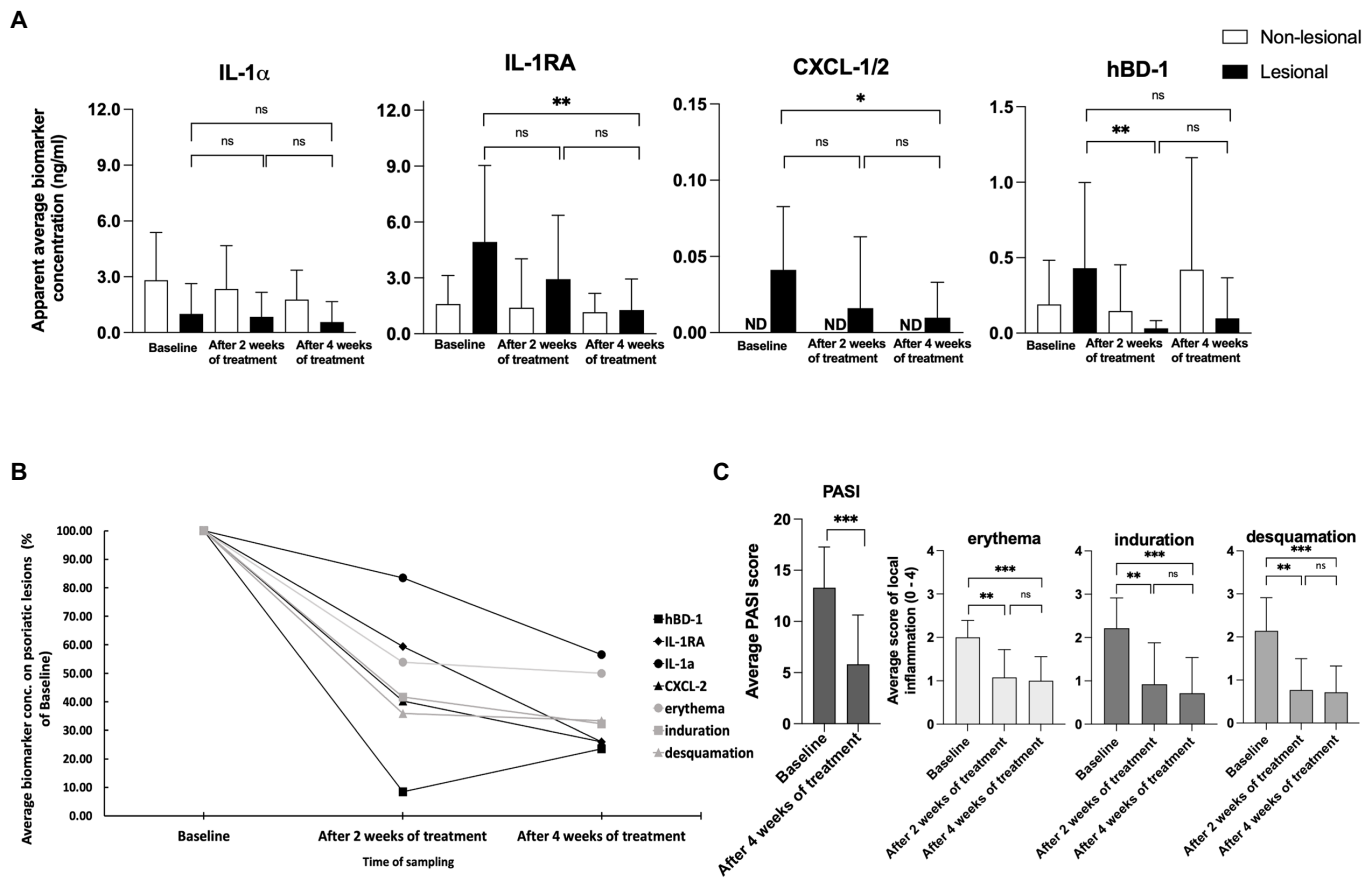
**(A)** Measurements of IL-1⍺, IL-1RA, CXCL-1/2, and hBD-1 on non-lesional and lesional skin of psoriasis patients during narrow band UVB treatment. Biomarker measurements were performed on healthy apparent and lesional skin before treatment, and after two and four weeks of treatment at the same skin site using FibroTx TAP. The apparent average biomarker concentration of non-lesional skin is plotted with white bars (panels A–D), and average biomarker measurements of lesional skin sites are presented with back bars (panels A–D). *Y*-axis: apparent concentration of IL-1α, IL-1RA, CXCL-1/2, and hBD-1 on the skin in ng/mL. *X*-axis: time point of biomarker sampling. Error bars in graphs A–D represent the standard deviations from the average of combined measurements of the patients (*N* = 14); ND, not detected. Statistical significance was determined with paired sample Wilcoxon signed-rank test (*p < 0.05, **p < 0.01, ***p < 0.001). **(B)** Measurements of IL-1⍺, IL-1RA, CXCL-1/2, and hBD-1 on lesional skin of psoriasis patients during narrow band UVB treatment. Combined average levels of biomarkers IL-1α, IL-1RA, CXCL-1/2, and hBD-1 sampled with TAP on lesional skin of psoriasis patient (*N* = 14) and scores of local erythema, induration, and desquamation of the same lesional site before and during treatment with narrow-band UVB combined with calcipotriol/betamethasone dipropionate ointment. Data presented as % of baseline values. **(C)** Changes in the psoriasis area severity index (PASI) and local scores for erythema, induration, and desquamation induced by narrow-band UVB treatment. The PASI score was documented before the treatment initiation and after 4 weeks of treatment. Local scores for erythema, induration, and desquamation were documented before the treatment initiation, and after two and four weeks of treatment at the same lesion sites. Each bar plotted in Figure 3A represents an average measurement of an analyzed clinical score of psoriasis patients (*N* = 14). Error bars on graphs present the standard deviations from the average of combined measurements of the patients (*N* = 14). Statistical significance was determined with paired sample Wilcoxon signed-rank test (*p < 0.05, **p < 0.01, ***p < 0.001).

During the 4 weeks course of UVB treatment, levels of IL-1α Did Not change on the lesional skin of psoriasis patients, but there was a modest decline In IL-1α on non-lesional skin (Figure 3). In contrast, levels of Pro-inflammatory IL-1RA (*p* < 0.01) and CXCL-1/2 (*p* < 0.05) showed a significant reduction in lesional skin in response to therapy. Over the four weeks of treatment, levels of IL-1RA on lesional skin reduced to a level similar to the amount of IL-1RA on non-lesional skin. No alterations were measured for IL-1RA on non-lesional skin during the course of treatment and CXCL-1/2 remained undetectable (Figure 3). The levels of antimicrobial peptide hBD-1 detected at baseline on non-lesional skin were nearly twofold lower compared to the levels captured on lesional skin. However, after four weeks of narrow-band UVB treatment, the levels of hBD-1 detected on non-lesional skin increased compared to the baseline with an approximately twofold increase, contrary to the levels of hBD-1 captured on lesional skin, which showed a nearly fourfold decrease compared to the amounts of baseline hBD-1 (Figure 3.). Kinetics of the downregulation of analyzed skin surface proteins showed a notable difference between the individual markers. Levels of IL-1α displayed a slower response reaching just 40% of reduction whereas IL-1RA and CXCL-1/2 were reduced by 75% and hBD-1 by 80% of their baseline levels on lesional skin after four weeks of treatment (Figure 3B).

### Skin surface biomarkers associated with disease activity scores over narrow-band ultraviolet B therapy

As a result of the narrow-band UVB treatment, the PASI score dropped an average of 57.71 percent during treatment, a highly significant difference (*p* < 0.001; Figure 3C). Local scores for erythema, induration, and desquamation showed high significant improvements of the lesions measured by FibroTx TAP (*p* < 0.01, *p* < 0.01, *p* < 0.01, respectively, Figure 3C).

IL-1α, IL-1RA, and CXCL-1/2 are cytokines directly involved in psoriasis skin inflammation (32, 35). Therefore we decided to assess whether these skin surface proteins measured by TAP correlated with disease severity over time. We analyzed the correlation between the values of IL-1α, IL-1RA, CXCL-1/2, and hBD-1 measurements from psoriatic skin against the PASI and the values of erythema, induration, and desquamation, at the area of the FibroTx TAP measurements. Data collected on the baseline and after 4 weeks of treatment were combined for Spearman rank correlation analysis positive association between skin surface proteins, PASI (*r* > 0.4), and the values of erythema, induration, and desquamation was observed over time for CXCL-1/2 and IL-1RA (*r* > 0.3) indicating higher levels of skin surface CXCL-1/2, IL-1RA for higher disease severity (Supplementary Table S6). Moderate correlation for skin surface anti-microbial hBD-1 and erythema (*r* = 0.4, *p* = 0.02), and induration (*r* = 0.39, *p* = 0.04) was observed. No correlation between IL-1α and disease severity scores over UVB therapy was noted.

Analyses of the IL-1RA over IL-1α ratio also confirmed the clinically observed pattern of normalization of skin in lesions measured. The ratio between IL-1RA and IL-1α, measured on lesions (ng/mL), declined from 4.89 to 2.25. In contrast, the ratio between IL-1RA and IL-1α, measured on non-lesional skin, remained stable, changing from 0.57 to 0.65 during treatment (Supplementary Table S7).

No adverse events were reported in the FibroTx TAP measurements, neither on non-lesional skin nor on lesional skin, and neither in patients nor in healthy individuals either by visual assessment (e.g., signs of redness) or upon inquiry (e.g., irritation, itching, and pain).

## Discussion and conclusion

This exploratory pilot study aimed to assess whether expression patterns of inflammatory proteins known to be involved in psoriasis measured non-invasively from the skin surface of adult psoriasis patients associated with the clinical scores for psoriasis severity and whether the changes in skin surface biomarker levels followed the clinical features in response to therapy. The choice for measuring skin surface proteins using the FibroTx TAP was because TAP is a non-invasive sampling technology that does not affect the skin, i.e., protein measurements are not biased by skin responding to the measurement method, and do not interfere with biological processes in and on the skin (28). Moreover, the method collects the analytes directly from the skin surface within one step and no additional extraction procedures after sampling are needed like in the case of the tape strip method (22).

The panel of IL-1α, IL-1RA, CXCL-1/2, and hBD-1 was chosen for the pilot study because these proteins have shown reliable quantitative and qualitative measurements from the skin surface using TAP (27, 29), and based on their role in inflammation of psoriasis reported in the literature. IL-1α and IL-1RA are examples of pro- and anti-inflammatory interleukins, respectively, that are known to play important roles in skin homeostasis and skin inflammation, including psoriasis (28, 32–35). The combination of chemokines CXCL-1 and -2 was chosen because of their role in attracting neutrophils to psoriatic skin lesions, which leads to T-cell activation (34, 36). Hence CXCL-1/2 is a valuable biomarker for monitoring inflammatory-related processes in the skin. Epithelial-produced antimicrobial peptide hBD-1 is detected also in immune cells, such as macrophages and monocytes (37), linking these signaling molecules with immune system regulation. Further, Uzuncakmak et al. monitored by invasive methods the alteration of tissue expression of human beta defensin-1 in PV following phototherapy (38), therefore presenting hBD-1 as an interesting candidate for non-invasive monitoring of its pattern in response to therapy.

Non-invasive skin surface measurements with TAP confirm that proteins such as IL-1RA, CXCL-1/2, and hBD-1 are present in higher amounts on lesional skin, whereas others, such as IL-α, are found in reduced amounts on lesional skin in comparison with non-lesional and healthy skin. This was supported by reports in the literature, which assessed these patterns through more invasive technologies, such as mRNA and immune histochemistry (IHC) analysis using skin biopsies and protein analysis (28, 34, 38–41). Therefore, it appears that non-invasive measurements of soluble proteins found on the skin, e.g., as measured by TAP, both qualitatively and quantitatively correlate with proteins found in the skin, as measured by invasive methods.

In the literature, there is ample evidence that IL-1α is found in decreased levels, and IL-1RA in increased levels in psoriatic lesional skin in comparison with non-lesional skin (32, 39, 40). TAP measurements of IL-1α and IL-1RA on the skin of adult psoriasis patients thus fit the bulk of evidence in the literature. One of the possible reasons for these levels of assessed IL-1α and IL-1RA on lesional versus non-lesional skin could be through skin barrier disruption, which is a distinguishing parameter for lesions. Preformed IL-1α is described to be stored in epidermis (42) and is released and depleted due to the skin barrier damage compared to the healthy tissue where the skin barrier is intact.

PV manifests itself in physical changes of the skin layers, such as thickening of the epidermis and the presence of a characteristic low-density layer between the epidermis and dermis, the so-called sub-epidermal low-echogenic band (SLEB) that can be measured *via* ultrasound (43, 44). Our measurements clearly showed a statistically significant thickening of the epidermis, SLEB, and dermis in lesional skin in comparison with non-lesional skin. A moderate positive correlation between CXCL-1/2 and SLEB thickness of lesional skin was observed. SLEB presents dermis-reduced echogenicity that is caused by edema and inflammatory cell infiltration at the inflamed lesions, which in turn can be explained by elevated levels of CXCL-1/2 produced by T-cells and keratinocytes in inflamed lesions. This connection between skin surface CXCL-1/2 and dermal SLEB suggests that local skin surface proteins can reflect the condition of the skin from deeper layers. Interestingly, neither ultrasound measurements nor visual assessments of lesional skin correlated in a highly statistically significant sense; only a mild correlation between skin induration and SLEB thickness was observed, and thus it appears that visual-, ultrasound- and protein measurements can quantify disease intensity.

We followed patients during short-wave UVB treatment to determine whether measurements of skin-surface IL-1α, IL-1RA, CXCL-1/2, and hBD-1 reflect disease intensity merely in a qualitative state, i.e., inflamed or not inflamed, or whether skin surface measurements of these biomarkers can reflect disease intensity quantitatively. Four weeks of narrow-band UVB treatment resulted in notable changes in skin surface protein levels (Figure 3) in addition to an improvement in disease severity scores (Figure 3C). Interestingly, the analyzed biomarkers displayed different treatment response kinetics – whereas IL-1RA, CXCL-1/2, and hBD-1 presented close to 80% reduction compared to baseline levels assessed on lesional skin, levels of IL-1α displayed slower and low response, reaching just 40% of reduction (Figure 3B) when compared to baseline levels. This distinction of IL-1α lower response kinetics could be explained by the importance of this cytokine in the regulation of innate immune defense mechanisms (45) and by the constant production of local keratinocytes. Therefore, pre-produced IL-1α could emerge from the depths of the epidermal layer whereas anti-inflammatory IL-1RA, synthesized previously in counter to inflammation, reduced in response to therapy. This hypothesis seems to be supported by the measurement of IL-1α and IL-1RA levels of the same psoriasis patients’ non-lesional skin. Response kinetics and the detected alteration in levels of IL-1α on lesional skin resembled the kinetics and the detected reduction rate of skin surface IL-1α on healthy apparent skin, whereas kinetics and decreased levels of IL-RA were more robust on psoriatic lesion when compared with measurements of non-lesional skin.

Interestingly, although skin surface hBD-1 stated higher levels on psoriatic lesions than non-lesional skin, followed by local scores for erythema, induration, and desquamation during UVB therapy, reverse levels of hBD-1 were detected on psoriasis lesions compared to patients’ non-lesional skin after 4 weeks of treatment. We hypothesized it could be related, on the one hand, to the integrity of the skin barrier host defense mechanism of healthy skin and, on the other hand, due to the apoptosis caused by narrow-band UVB of local keratinocytes producing hBD-1 in psoriasis lesions.

Although correlations between skin surface protein levels and disease severity were observed, it was interesting that analysis of skin surface biomarker levels assessed with FibroTx TAP revealed that skin surface measurements of IL-1RA and CXCL-1/2 displayed a different pattern than achieved by visual evaluation of local inflammation (Figure 3C). Visual assessment for erythema, induration, and desquamation decreased after 2 weeks of treatment and displayed similar levels after 4 weeks of treatment, whereas IL-1RA and CXCL-1/2 normalized more gradually through therapy (Figure 3). This confirmed that measuring the ‘molecular root’ of inflammation appears to have value as an objective, non-invasive biomarker measurement for scoring disease intensity. The difference in kinetics of analyzed biomarkers suggested that changes in skin surface proteins are not a uniform reflection of skin healing, but rather reflect individual changes of expression in the skin. Nevertheless, the patient cohort of the current study was limited, and a study with a larger cohort of patients is needed for firm conclusions.

In conclusion, we could measure IL-1α, IL-1RA, CXCL-1/2, and hBD-1 on the skin of psoriasis patients, with clear differences between lesional and non-lesional skin, which supports the hypothesis that TAP is an applicable tool for non-invasive skin surface protein measurement on skin. Further, correlating skin surface measurements of analyzed proteins on the skin from psoriasis patients with clinical assessments of psoriasis severity suggests that these protein measurements may have potential as biomarkers. Nonetheless, a substantially larger study will be necessary to further validate measurements of IL-1α, IL-1RA, CXCL-1/2, and hBD-1 on the skin of psoriasis patients for diagnostic, prognostic, and therapeutic biomarker applications. Whether the conclusions about the applicability of the FibroTx TAP method and analyzed biomarkers can be applied to other skin diseases, skin surface biomarkers, and/or treatment methods is not known and will require additional research.

## Data availability statement

The raw data supporting the conclusions of this article will be made available by the authors, without undue reservation.

## Ethics statement

This research was conducted in accordance with the World Medical Association Declaration of Helsinki. Ethical approval for the studies is covered by Decision No. 2551 from the Tallinn Medical Research Ethical Committee. The Declaration of Helsinki protocols were followed. The patients/participants provided their written informed consent to participate in this study.

## Author contributions

KO analyzed the data. KO, KK, and PS wrote the manuscript. KK, KA, MK, KO, and KS conducted TAP biomarker measurements from the skin of psoriasis patients. KO and KS designed, and KO, KS, and JA performed experiments related to TAP biomarker measurement performed on of skin of healthy volunteers. KA and AM performed ultrasound measurements. TN, KK, and PS were responsible for the overall study design. All authors have read and approved the final manuscript.

## Funding

This study received funding from FibroTx LLC. The study was supported by the Estonian Research Council as personal research Grant PUT1465 and Estonian Research Council Team grant PRG1189 to KK, and the publication fee will be covered by the Estonian Research Council Team grant PRG1189 to KK.

## Conflict of interest

KO, KS, JA, AM, and TN are all employed by the company FibroTx. TN is a founder and shareholder of FibroTx. PS is a consultant at FibroTx and employed by PS! Pharmaconsult.

The remaining authors declare that the research was conducted in the absence of any commercial or financial relationships that could be construed as a potential conflict of interest.

This study received funding from FibroTx LLC. FibroTx LLC had the following involvement with the study: overseeing the study design and providing the FibroTx Transdermal analysis Patches.

## Publisher’s note

All claims expressed in this article are solely those of the authors and do not necessarily represent those of their affiliated organizations, or those of the publisher, the editors and the reviewers. Any product that may be evaluated in this article, or claim that may be made by its manufacturer, is not guaranteed or endorsed by the publisher.
