# Supplementary material for: Assessment of soluble skin surface protein levels for monitoring psoriasis vulgaris in adult psoriasis patients using non-invasive transdermal analysis patch: A pilot study
Source: Front Med (Lausanne). 2023 Mar 2;10:1072160. doi: 10.3389/fmed.2023.1072160 (PMC10019527; doi:10.3389/fmed.2023.1072160)
Supplement: Supplementary file 1 [file Table_1.DOCX]

**Supplementary Table S1**

|  | **Mean ng/ml** | | **Ratio of IL-1RA/IL-1⍺** | | | |
| --- | --- | --- | --- | --- | --- | --- |
|  |  |  |  |  | **Molar ratio** | |
| **Sampling site** | **IL-1⍺** | **SD** | **IL-1RA** | **SD** | **Precursor** | **Mature** |
| **Healthy skin** | 3.14 | ± 0.74 | 0.86 | ±0.18 | 0.50 | 0.29 |
| **Non-lesional skin** | 2.16 | ± 2.44 | 0.93 | ±0.88 | 0.77 | 0.45 |
| **Lesional skin** | 0.98 | ±1.44 | 2.05 | ±1.89 | 3.77 | 2.19 |

**Table S1. Ratio of IL-1RA over IL-1α on the skin of healthy volunteers and on the skin of psoriasis patients.** The average concentration of IL-1α and IL-1RA on normal skin of healthy volunteers (N = 10), non-lesional and lesional skin of psoriasis patients (N = 30) is presented in Table 1 in ng/ml. The standard deviation (± SD) presented in table present the standard deviation from average of combined measurements of the 10 healthy volunteers and 30 psoriasis patients, respectively. Additionally, molar ratio of IL-1RA over precursor and mature IL-1α (Ratio of IL-1RA/IL-1α) is presented.

**Supplementary Table 2A**

| **Analyte** | **Spearman correlation coefficient** | **IL-1α** | **IL-1RA** | **CXCL 1/2** | **hBD-1** | **Epidermis thickness** | **Dermis thickness** |
| --- | --- | --- | --- | --- | --- | --- | --- |
| **Epidermis thickness** | Spearman's rho | 0.122 | 0.017 | -0.349 | -0.086 | — |  |
|  | p-value | 0.52 | 0.93 | 0.059 | 0.651 | — |  |
| **Dermis thickness** | Spearman's rho | 0.015 | 0.023 | 0.081 | 0.107 | 0.027 | — |
|  | p-value | 0.939 | 0.902 | 0.669 | 0.574 | 0.886 | — |
| **SLEB thickness** | Spearman's rho | 0.238 | **0.45*** | 0.285 | 0.177 | 0.074 | -0.095 |
|  | p-value | 0.205 | **0.013** | 0.127 | 0.35 | 0.699 | 0.619 |

**Supplementary Table 2 A. Correlation analysis between FibroTx TAP measurements of IL-1α, IL-1RA, CXCL-1/2 and hBD-1 on non-lesional skin of psoriasis patients combined with ultrasound measurements of epidermis-, dermis- and SLEB thickness at the same analysis site.** Correlation between biomarker measurements and skin layer thickness of psoriasis patients (N = 30) was assessed using Spearman’s rank correlation analysis. Statistical significances were verified with probability value (*p*-value). Relevant correlations are flagged with asterisk (** p* < *0.05, ** p* < *0.01, *** p* < *0.001*)*.*

**Supplementary Table 2B**

| **Analyte** | **Spearman correlation coefficient** | **IL-1α** | **IL-1RA** | **CXCL 1/2** | **hBD-1** | **Epidermis thickness** | **Dermis thickness** |
| --- | --- | --- | --- | --- | --- | --- | --- |
| **Epidermis thickness** | Spearman's rho | 0.086 | 0.015 | -0.192 | -0.214 | — |  |
|  | p-value | 0.653 | 0.936 | 0.309 | 0.257 | — |  |
| **Dermis thickness** | Spearman's rho | -0.112 | -0.128 | 0.048 | -0.108 | 0.145 | — |
|  | p-value | 0.554 | 0.5 | 0.799 | 0.572 | 0.446 | — |
| **SLEB thickness** | Spearman's rho | 0.13 | 0.296 | **0.512**** | 0.359 | 0.138 | 0.079 |
|  | p-value | 0.494 | 0.112 | **0.004** | 0.051 | 0.467 | 0.678 |

**Supplementary Table 2B. Correlation analysis between FibroTx TAP measurements of IL-1α, IL-1RA, CXCL-1/2 and hBD-1 on lesional skin of psoriasis patients and between ultrasound measurements of epidermis-, dermis- and SLEB thickness at the same analysis site.** Correlation between biomarker measurements and skin layer thickness of psoriasis patients (N = 30) was assessed using Spearman’s rank correlation analysis. Statistical significances were verified with probability value (*p*-value). Relevant correlations are flagged with asterisk (** p* < *0.05, ** p* < *0.01, *** p* < *0.001*)*.*

**Supplementary Table 2C**

| **Analyte** | **Spearman correlation coefficient** | **PASI** | **Induration** | **Desquama-tion** | **Erythema** | **Epidermis thickness** | **Dermis thickness** |
| --- | --- | --- | --- | --- | --- | --- | --- |
| **Epidermis thickness** | Spearman's rho | -0.164 | -0.225 | -0.181 | 0.01 | — |  |
|  | p-value | 0.388 | 0.231 | 0.34 | 0.956 | — |  |
| **Dermis thickness** | Spearman's rho | 0.105 | 0.112 | 0.039 | -0.03 | 0.145 | — |
|  | p-value | 0.583 | 0.556 | 0.838 | 0.874 | 0.446 | — |
| **SLEB thickness** | Spearman's rho | 0.241 | **0.402*** | 0.339 | 0.36 | 0.138 | 0.079 |
|  | p-value | 0.199 | **0.028** | 0.067 | 0.051 | 0.467 | 0.678 |

**Supplementary Table 2C. Correlation analysis between local clinical scores and epidermis-, dermis- and SLEB thickness measured from lesional skin by ultrasound.** Correlation between local clinical scores and epidermal-, dermal- and SLEB thickness measured from lesional skin of psoriasis patients (N = 30) was assessed using Spearman’s rank correlation analysis. Statistical significances were verified with probability value (*p*- value). Relevant correlations are flagged with asterisk. The FibroTx TAP measurements, clinical scores and ultrasound measurements were performed all at the exact same skin lesion (** p* < *0.05, ** p* < *0.01, *** p* < *0.001).*

**Supplementary Table 3**

| **Analyte** | **Spearman correlation coefficient** | **IL-1α** | **IL-1RA** | **CXCL-1/2** | **hBD-1** |
| --- | --- | --- | --- | --- | --- |
| **PASI** | Spearman's rho | 0.040 | **0.424*** | **0.518**** | **0.436*** |
|  | p-value | 0.838 | 0.024 | 0.004 | 0.020 |
| **Erythema** | Spearman's rho | 0.111 | **0.56**** | **0.38*** | **0.422*** |
|  | p-value | 0.572 | 0.001 | 0.046 | 0.025 |
| **Induration** | Spearman's rho | 0.036 | **0.380*** | **0.533**** | **0.390*** |
|  | p-value | 0.855 | 0.0459 | 0.003 | 0.040 |
| **Desquamation** | Spearman's rho | 0.074 | **0.398*** | **0.54**** | 0.275 |
|  | p-value | 0.709 | 0.035 | 0.003 | 0.156 |

**Supplementary Table 3. Analysed correlations of FibroTx TAP measurements of IL-1α, IL-1RA, CXCL-1/2 and hBD-1 on lesional skin between PASI and local score of erythema, induration, and desquamation in psoriasis patients over UVB therapy.** Data collected on baseline and after 4 weeks of UVB treatment was combined for Spearman rank correlation analysis. Statistical significances were verified with probability value (*p* - value). Relevant correlations are flagged with asterisk (** p* < *0.05, ** p* < *0.01, *** p* < *0.001*)*.*

**Supplementary Table 4**

|  |  |  | **Mean ng/ml of IL-1⍺ and IL-1RA** | | |  |  |  | **Molar ratio of IL-1RA/IL-1⍺** | | | |
| --- | --- | --- | --- | --- | --- | --- | --- | --- | --- | --- | --- | --- |
|  | **Non-lesional** | | **Lesional** | | **Non-lesional** | | **Lesional** | | **Precursor** | | **Mature** | |
| **Sampling time** | **IL-1⍺** | **SD** | **IL-1⍺** | **SD** | **IL-1RA** | **SD** | **IL-1RA** | **SD** | **NL** | **L** | **NL** | **L** |
| **Baseline** | 2.82 | ± 2.56 | 1.01 | ± 1.63 | 1.60 | ±1.54 | 4.94 | ±4.10 | 1.10 | 8.89 | 0.64 | 5.16 |
| **After 2 weeks of treatment** | 2.34 | ±2.33 | 0.84 | ±1.32 | 1.41 | ±2.63 | 2.93 | ±3.42 | 1.17 | 6.32 | 0.68 | 3.67 |
| **After 4 weeks of treatment** | 1.78 | ±1.57 | 0.57 | ±1.09 | 1.16 | ±1.01 | 1.28 | ±1.66 | 1.41 | 4.08 | 0.82 | 2.37 |

**Supplementary Table 4. Ratios between IL-1RA and IL-1α on non-lesional and lesional skin of psoriasis patients.** The mean concentration (ng/ml) of IL-1α and IL-1RA sampled on lesional (L) and non-lesional (NL) skin of psoriasis patients (N = 14) before treatment initiation (baseline), after two weeks and after four weeks of treatment is presented in Table 4. The standard deviation (SD) in table presents the standard deviation from average of combined measurements of psoriasis patient NL and L skin site, respectively. Additionally, molar ratio of IL-1RA over precursor and mature IL-1α (ratio of IL-1RA/IL-1α) is presented.
