# Supplementary material for: Assessment of soluble skin surface protein levels for monitoring psoriasis vulgaris in adult psoriasis patients using non-invasive transdermal analysis patch: A pilot study
Source: Front Med (Lausanne). 2023 Mar 2;10:1072160. doi: 10.3389/fmed.2023.1072160 (PMC10019527; doi:10.3389/fmed.2023.1072160)
Supplement: Supplementary file 2 [file Data_Sheet_1.PDF]

Supplementary Materials

Content

Materials and Methods 2

Transdermal Analysis Patch 2

Composition of the Transdermal Analysis Patch.. 2

Antibodies used for TAP 3

Figures.. 4

Figure S1. Measurements of IL-1α, IL-1RA, CXCL-1/2, and hBD-1 on non-lesional and lesional skin of psoriasis patients using FibroTx TAP. 4

Participant characteristics 5

Characteristics of healthy volunteers.. 5

Supplementary Table S1. Characteristics of the recruited healthy volunteers (N = 10).. 5

Characteristics of recruited psoriasis vulgaris patients 6

Supplementary Table S2. Characteristics of the recruited patients (N = 30).. 6

Supplementary Table S3. Characteristics of the patients subjected to narrow-band UVB treatment (N = 14) 7

Tables 8

Supplementary Table S4. The ratio of IL-1RA over IL-1α on the skin of healthy volunteers and on the skin of psoriasis patients.. 8

Supplementary Table S5A. Correlation analysis between FibroTx TAP measurements of IL-1α, IL-1RA, CXCL-1/2, and hBD-1 on non-lesional skin of psoriasis patients combined with ultrasound measurements of the epidermis-, dermis- and SLEB, thickness at the same analysis site. 9

Supplementary Table S5B. Correlation analysis between FibroTx TAP measurements of IL-1α, IL-1RA, CXCL-1/2, and hBD-1 on lesional skin of psoriasis patients and between ultrasound measurements of the epidermis-, dermis- and SLEB thickness at the same analysis site. 9

Supplementary Table S5C. Correlation analysis between local clinical scores and epidermis-, dermis- and SLEB thickness measured from lesional skin by ultrasound.. 10

Supplementary Table S6. Analysed correlations of FibroTx TAP measurements of IL-1α, IL-1RA, CXCL-1/2, and hBD-1 on lesional skin between PASI and a local score of erythema, induration, and desquamation in psoriasis patients over UVB therapy. 11

6Supplementary Table S7. Ratios between IL-1RA and IL-1 α on non-lesional and lesional skin of psoriasis patients. 11

Materials and Methods

Transdermal Analysis Patch

Composition of the Transdermal Analysis Patch

Transdermal Analyses Patch (TAP) consists of a micro-array that is supported by a dermal adhesive plaster (3M White Nonwoven Medical Tape, 9907HTW) for easy fixture to skin. In between the antibody microarray and the plaster, a layer is positioned that serves as a fluid reservoir for the buffer needed for protein capturing from the skin. In addition, this expandable layer serves as a pressure pad to ensure close contact of the microarray to the skin. Each TAP micro-array contains two spots of positive controls (IgG; Goat anti-human IgG Lab AS, Estonia) to determine the specificity of biomarker measurements, and a panel of capturing antibodies, of which each capturing antibody variant is printed in triplicate (N = 3), as discrete spots on the membrane (GE HealthCare, 10600002) by non-contact dispensing (BioDot AD3400 printer). The concentrations of capturing antibodies used for TAP capturing antibody micro-arrays were as follows: 0.25 ng of anti-IL-1α, 2.25 ng of anti-IL-1RA, 2.25 ng of anti- CXCL- 1/2 and 2.25 ng of anti-hBD-1 per spot, additionally each micro-array contained a negative control (PBS - with 20% (v/v) glycerol) and positive control (0.03 ng biotinylated anti- hBD-1). Captured proteins are analysed, both qualitatively and quantitatively, on the antibody micro-array using spot-ELISA.

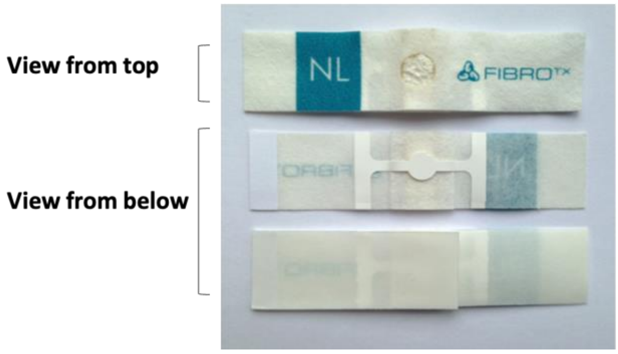

Image S1 Composition of the Transdermal Analyses Patch (TAP). TAP consists of a plaster with a nitrocellulose core that contains the capture antibody microarray clearly visible in the middle.

Visualisation of captured proteins using spot-ELISA

48-well plates (Greiner BioOne) for assay were blocked with 1% BSA (w/v) in PBS (pH = 7.4). Subsequently, wells were washed with milli-Q water and dried. For further processing TAP capture antibody micro-arrays were placed into blocked 48-well plates and wetted with PBS. To create standard

curves, capture antibody microarrays were incubated for 20 minutes at 33°C with a mixture of recombinant proteins diluted in PBS + 0.05% (v/v) Tween-20. Unbound proteins were washed from the membrane with a wash buffer.

To determine the concentrations of skin surface captured biomarkers antibody capturing micro-arrays incubated on the skin were removed from plaster and placed into blocked 48-well plates and washed using wash buffer. Antibody capturing micro-arrays subjected to standard curves and skin surface biomarker analysis were blocked for 20 min at room temperature in 5% BSA (w/v) in PBS (pH = 7.4). A biotinylated secondary antibody was added to each capturing antibody microarray and incubated for 45 min at room temperature. The Catalysed Signal Amplification (CSA) System (Dako, K-1497) was used for signal amplification. Tyramide and Anti-Fluorescein-HRP solution was diluted in diluent (PBS + 0.05% (v/v) Tween-20) to 10% and micro-arrays were incubated for 15 min at room temperature. For signal visualisation, Substrate-Chromogen solution diluted to 0.4% in Substrate Buffer Concentrate was used, and micro-arrays were incubated for 15 min at room temperature. The reaction was stopped with milli-Q water. Signals of captured biomarkers were quantified by comparing the signals of these proteins captured from the skin of subjects using FibroTx TAP capture antibody micro-array incubated with fixed amounts of recombinant proteins.

Antibodies used for TAP

Human GRO-ß (CXCL-2) ELISA Development Kit (Cat. No: 900-K120, PeproTech), Human IL-1α ELISA Development Kit (Cat. No: 900-K11, PeproTech), Human hBD-1 ELISA Development Kit (Cat. No: 900-K202, PeproTech), Human IL-1RA ELISA Development Kit (Cat. No: 900-K474, PeproTech).

Figures

Figure S1. Measurements of IL-1α, IL-1RA, CXCL-1/2, and hBD-1 on non-lesional and lesional skin of psoriasis patients using FibroTx TAP.

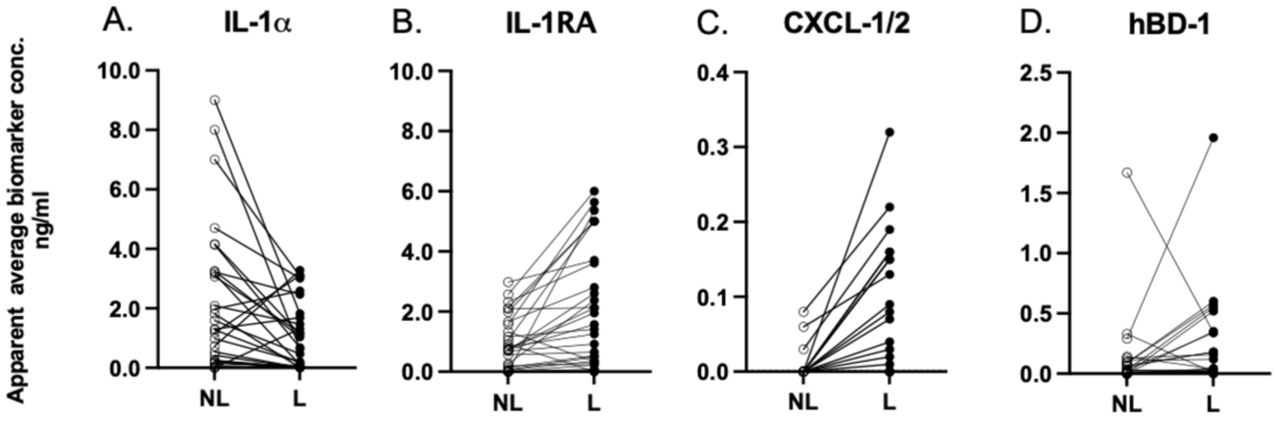

Figure S1. Measurements of IL-1α, IL-1RA, CXCL-1/2, and hBD-1 on non-lesional and lesional skin of psoriasis patients using FibroTx TAP. In panels A - D single measurements of IL-1α, IL-1RA, CXCL-1/2 and hBD-1detected from non-lesional (NL) skin and lesional skin (L) of 30 psoriasis patients have been depicted, each line represents a single patient. Y-axis: Apparent concentration of analysed biomarker on the skin in ng/ml, X-axis: sampling site.

Participant characteristics

Characteristics of healthy volunteers

The healthy volunteers who were enrolled between 2015 -2016 as a part of this study were unrelated Caucasians living in Estonia. The mean ± standard deviation of the age of healthy participants was 50.4 ± 8.47 years, respectively. Of 10 subjects 3 (30%) were male and 7 were female (70%) (Supplementary Table S1).

Supplementary Table S1. Characteristics of the recruited healthy volunteers (N = 10)

Sex	Age	Body site
M	49	arm
F	49	arm
F	51	arm
M	61	arm
F	58	arm
F	46	arm
F	59	arm
M	33	arm
F	43	arm
F	55	arm

Characteristics of recruited psoriasis vulgaris patients

The patients enrolled as part of this study were unrelated Caucasians living in Estonia and were recruited from the outpatient clinic between 2015 – 2016 as they arrived for the appointment, with no discrimination with regard to the clinical characteristics of the disease. The mean ± standard deviation (SD) of the age of patients was 45.65 ± 13.47, respectively. Of 30 patients 20 were male and 10 were female (Supplementary Table S2).

Supplementary Table S2. Characteristics of the recruited patients (N = 30)

						Body site	
**Sex**	**Age**	**PASI**	**Erythema**	**Induration**	**Desquamation**	**L**	**NL**
M	49	21.5	2	2	2	side	shoulder
M	51	18.4	2	2	3	buttock	back
M	44	27	2	1	2	forearm	forearm
M	23	15.8	3	2	2	lower back	lower back
M	37	12	2	1	2	lower side	lower back
M	22	17.8	3	2	2	lower back	back
F	31	20	3	1	4	abdomen	abdomen
M	39	21.9	3	2	3	buttock	buttock
M	45	13.4	2	2	2	buttock	lower back
F	45	13.5	2	1	2	side	side
M	24	14.8	1	2	2	abdomen	sternum
M	48	19.5	4	3	3	lower back	back
F	39	5	2	1	2	abdomen	arm
F	59	3	2	4	3	foot	foot
F	50	2	1	1	1	leg	leg
M	51	17.1	2	2	2	lower	lower back
M	40	4	2	1	2	elbow	lower back
M	71	8.5	2	1	2	leg	leg
F	58	4	1	1	1	sole	forearm
F	36	21.8	2	2	1	sole	forearm
F	50	28	3	3	1	leg	forearm
M	60	14.6	2	2	2	forearm	forearm
M	45	27.3	3	3	2	leg	foot
M	49	30.8	3	3	3	thigh	thigh
M	47	23.8	3	3	2	leg	leg
M	56	10.3	2	2	2	sole	leg
M	57	25.8	2	2	2	elbow	elbow
F	73	6	2	1	1	leg	leg
M	56	24.8	2	2	2	knee	leg
F	40	12.2	2	2	1	thigh	thigh

Supplementary Table S2. Characteristics of the recruited patients. Disease severity was quantified as Psoriasis Area and Severity Index (PASI) score and as local scores for erythema (0 – 4), induration (0 – 4), and desquamation (0 – 4). F- female; M – male; L -psoriatic lesion, NL – non-lesional skin site of the psoriasis patient.

Further, fourteen adult psoriasis vulgaris patients were included for monitoring skin surface biomarkers, PASI index, and local scores for erythema, inflammation, and scaling in response to narrow-band UVB treatment in combination with calcipotriol/betamethasone dipropionate ointment (Dovobet®) daily. The mean ± standard deviation (SD) of the age of patients was 45.64 (± 15), respectively. Of 14 patients 6 were male and 8 were female (Supplementary Table S3).
For all PV patients, disease severity was quantified as Psoriasis Area and Severity Index (PASI) score and local scores for erythema, induration, and desquamation. Detailed characteristics of recruited patients are presented in Supplementary Table S2 and S3.

Supplementary Table S3. Characteristics of the patients subjected to narrow-band UVB treatment (N = 14)

		**PASI**		**Erythema**			**Induration**			**Desquamation**			**Body site**	
**Sex**	**Age**	Baseline	After 4 weeks of treatment	Baseline	After 2 weeks of treatment	After 4 weeks of treatment	Baseline	After 2 weeks of treatment	After 4 weeks of treatment	Baseline	After 2 weeks of treatment	After 4 weeks of treatment	L	NL
F	55	9.9	4	2	1	1	1	0	0	2	1	1	leg	leg
M	51	8.1	2.4	2	1	0	2	0	0	2	1	0	back	back
F	52	10.7	4.6	2	1	1	2	1	1	1	1	1	leg	leg
M	35	13.2	2	2	1	1	2	2	0	2	2	1	leg	leg
M	27	14.4	2	2	1	1	2	1	1	2	1	1	leg	leg
F	51	14.5	16	2	1	1	2	1	1	2	1	1	arm	arm
M	57	18	11	2	1	1	2	1	2	1	0	1	arm	arm
F	50	7.8	4.4	2	1	1	2	0	1	2	0	0	elbow	arm
F	76	16	11.3	3	1	2	3	2	2	3	2	2	abdomen	abdomen
F	34	20.8	1.8	1	0	1	4	1	0	4	0	0	leg	leg
M	21	13.5	3	2	1	1	2	0	0	2	0	0	back	abdomen
F	52	12.2	7	2	3	2	3	3	2	3	1	1	elbow	arm
M	52	9	0	2	*	0	2	*	0	2	*	0	arm	arm
F	26	18	11.7	2	1	1	2	0	0	2	0	1	arm	arm

Supplementary Table S3. Characteristics of the patient subjected to narrow-band UVB treatment. Disease severity was quantified as Psoriasis Area and Severity Index (PASI) score and as local scores for erythema (0 – 4), induration (0 – 4), and desquamation (0 – 4). F- female; M – male; L -psoriatic lesion, NL – non-lesional skin site of the psoriasis patient.

Tables

Supplementary Table S4. The ratio of IL-1α over IL-1α on the skin of healthy volunteers and on the skin of psoriasis patients.

	Mean ng/ml				Ratio of IL-1RA/IL-1α	
					Molar ratio	
**Sampling site**	**IL-1α**	**SD**	**IL-1RA**	**SD**	**Precursor**	**Mature**
**Healthy skin**	3.14	± 0.74	0.86	±0.18	0.50	0.29
**Non-lesional skin**	2.16	± 2.44	0.93	±0.88	0.77	0.45
**Lesional skin**	0.98	±1.44	2.05	±1.89	3.77	2.19

Table S4. The ratio of IL-1RA over IL-1α on the skin of healthy volunteers and on the skin of psoriasis patients. The average concentration of IL-1α and IL-1RA on normal skin of healthy volunteers (N = 10), non-lesional and lesional skin of psoriasis patients (N = 30) is presented in Table 1 in ng/ml. The standard deviation (± SD) presented in the table presents the standard deviation from the average of combined measurements of the 10 healthy volunteers and 30 psoriasis patients, respectively. Additionally, a molar ratio of IL-1RA over precursor and mature IL-1α (Ratio of IL-1RA/IL-1α) is presented.

Supplementary Table S5A. Correlation analysis between FibroTx TAP measurements of IL-1α, IL-1RA, CXCL-1/2, and hBD-1 on non-lesional skin of psoriasis patients combined with ultrasound measurements of the epidermis-, dermis- and SLEB, thickness at the same analysis site.

Analyte	Spearman correlation coefficient	IL-1α	IL-1RA	CXCL 1/2	hBD-1	Epidermis thickness	Dermis thickness
**Epidermis thickness**	Spearman's rho	0.122	0.017	-0.349	-0.086	—	
	p-value	0.52	0.93	0.059	0.651	—	
**Dermis thickness**	Spearman's rho	0.015	0.023	0.081	0.107	0.027	—
	p-value	0.939	0.902	0.669	0.574	0.886	—
**SLEB thickness**	Spearman's rho	0.238	**0.45***	0.285	0.177	0.074	-0.095
	p-value	0.205	**0.013**	0.127	0.35	0.699	0.619

Supplementary Table S5 A. Correlation analysis between FibroTx TAP measurements of IL-1α, IL-1RA, CXCL-1/2, and hBD-1 on non-lesional skin of psoriasis patients combined with ultrasound measurements of epidermis-, dermis- and SLEB, thickness at the same analysis site. The correlation between biomarker measurements and the skin layer thickness of psoriasis patients (N = 30) was assessed using Spearman's rank correlation analysis. Statistical significances were verified with probability value (*p*-value). Relevant correlations are flagged with asterisk (* $p < 0.05$, ** $p < 0.01$, *** $p < 0.001$).

Supplementary Table S5B. Correlation analysis between FibroTx TAP measurements of IL-1α, IL-1RA, CXCL-1/2, and hBD-1 on lesional skin of psoriasis patients and between ultrasound measurements of the epidermis-, dermis- and SLEB thickness at the same analysis site.

Analyte	Spearman correlation coefficient	IL-1α	IL-1RA	CXCL 1/2	hBD-1	Epidermis thickness	Dermis thickness
**Epidermis thickness**	Spearman's rho	0.086	0.015	-0.192	-0.214	—	
	p-value	0.653	0.936	0.309	0.257	—	
**Dermis thickness**	Spearman's rho	-0.112	-0.128	0.048	-0.108	0.145	—
	p-value	0.554	0.5	0.799	0.572	0.446	—
**SLEB thickness**	Spearman's rho	0.13	0.296	**0.512****	0.359	0.138	0.079
	p-value	0.494	0.112	**0.004**	0.051	0.467	0.678

Supplementary Table S5B. Correlation analysis between FibroTx TAP measurements of IL-1α, IL-1RA, CXCL-1/2, and hBD-1 on lesional skin of psoriasis patients and between ultrasound measurements of the epidermis-, dermis- and SLEB thickness at the same analysis site. The correlation between biomarker measurements and skin layer thickness of psoriasis patients (N = 30) was assessed using Spearman's rank correlation analysis. Statistical significance were verified with probability value (*p*-value). Relevant correlations are flagged with an asterisk (* $p < 0.05$, ** $p < 0.01$, *** $p < 0.001$).

Supplementary Table S5C. Correlation analysis between local clinical scores and epidermis-, dermis- and SLEB thickness measured from lesional skin by ultrasound.

Analyte	Spearman correlation coefficient	PASI	Induration	Desquama-tion	Erythema	Epidermis thickness	Dermis thickness
**Epidermis thickness**	Spearman's rho	-0.164	-0.225	-0.181	0.01	—	
	p-value	0.388	0.231	0.34	0.956	—	
**Dermis thickness**	Spearman's rho	0.105	0.112	0.039	-0.03	0.145	—
	p-value	0.583	0.556	0.838	0.874	0.446	—
**SLEB thickness**	Spearman's rho	0.241	**0.402***	0.339	0.36	0.138	0.079
	p-value	0.199	**0.028**	0.067	0.051	0.467	0.678

Supplementary Table S5C. Correlation analysis between local clinical scores and epidermis-, dermis- and SLEB thickness measured from lesional skin by ultrasound. The correlation between local clinical scores and epidermal-, dermal- and SLEB thickness measured from lesional skin of psoriasis patients (N = 30) was assessed using Spearman's rank correlation analysis. Statistical significance was verified with probability value (*p*-value). Relevant correlations are flagged with an asterisk. The FibroTx TAP measurements, clinical scores, and ultrasound measurements were performed all at the exact same skin lesion (* $p < 0.05$, ** $p < 0.01$, *** $p < 0.001$).

Supplementary Table S6. Analysed correlations of FibroTx TAP measurements of IL-1α, IL-1RA, CXCL-1/2, and hBD-1 on lesional skin between PASI and a local score of erythema, induration, and desquamation in psoriasis patients over UVB therapy.

Analyte	Spearman correlation coefficient	IL-1α	IL-1RA	CXCL-1/2	hBD-1
**PASI**	Spearman's rho	0.040	**0.424***	**0.518****	**0.436***
	p-value	0.838	0.024	0.004	0.020
**Erythema**	Spearman's rho	0.111	**0.56****	**0.38***	**0.422***
	p-value	0.572	0.001	0.046	0.025
**Induration**	Spearman's rho	0.036	**0.380***	**0.533****	**0.390***
	p-value	0.855	0.0459	0.003	0.040
**Desquamation**	Spearman's rho	0.074	**0.398***	**0.54****	0.275
	p-value	0.709	0.035	0.003	0.156

Supplementary Table S6. Analysed correlations of FibroTx TAP measurements of IL-1α, IL-1RA, CXCL-1/2, and hBD-1 on lesional skin between PASI and a local score of erythema, induration, and desquamation in psoriasis patients over UVB therapy. Data collected on the baseline and after 4 weeks of UVB treatment was combined for Spearman rank correlation analysis. Statistical significance was verified with probability value (*p*-value). Relevant correlations are flagged with an asterisk (* $p < 0.05$, ** $p < 0.01$, *** $p < 0.001$).

Supplementary Table S7. Ratios between IL-1RA and IL-1 α on non-lesional and lesional skin of psoriasis patients.

	Mean ng/ml of IL-1α and IL-1RA								Molar ratio of IL-1RA/IL-1α			
	**Non-lesional**		**Lesional**		**Non-lesional**		**Lesional**		**Precursor**		**Mature**	
**Sampling time**	**IL-1α**	**SD**	**IL-1α**	**SD**	**IL-1RA**	**SD**	**IL-1RA**	**SD**	**NL**	**L**	**NL**	**L**
**Baseline**	2.82	± 2.56	1.01	± 1.63	1.60	±1.54	4.94	±4.10	1.10	8.89	0.64	5.16
**After 2 weeks of treatment**	2.34	±2.33	0.84	±1.32	1.41	±2.63	2.93	±3.42	1.17	6.32	0.68	3.67
**After 4 weeks of treatment**	1.78	±1.57	0.57	±1.09	1.16	±1.01	1.28	±1.66	1.41	4.08	0.82	2.37

Supplementary Table S7. Ratios between IL-1RA and IL-1α on non-lesional and lesional skin of psoriasis patients. The mean concentration (ng/ml) of IL-1α and IL-1RA sampled on lesional (L) and non-lesional (NL) skin of psoriasis patients (N = 14) before treatment initiation (baseline), after two weeks and after four weeks of treatment is presented in Table 4. The standard deviation (SD) in the table presents the standard deviation from the average of combined measurements of psoriasis patient NL and L skin site, respectively. Additionally, a molar ratio of IL-1RA over precursor and mature IL-1α (ratio of IL-1RA/IL-1α) is presented.
