# Supplementary figures and images for: Assessment of soluble skin surface protein levels for monitoring psoriasis vulgaris in adult psoriasis patients using non-invasive transdermal analysis patch: A pilot study
Source: Front Med (Lausanne). 2023 Mar 2;10:1072160. doi: 10.3389/fmed.2023.1072160 (PMC10019527; doi:10.3389/fmed.2023.1072160)

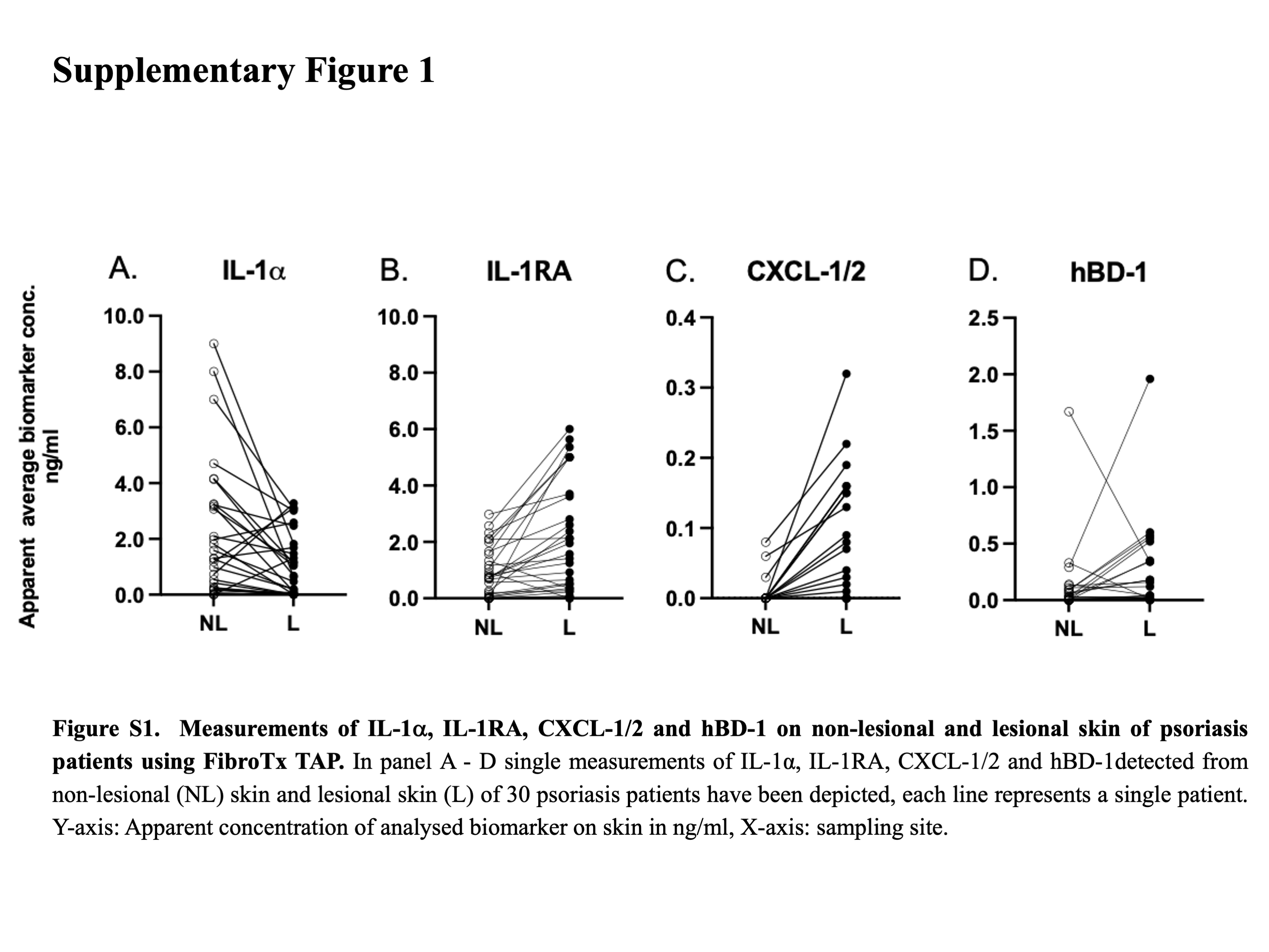

Supplement: Supplementary file 3 [file Image_1.TIFF]
